# Retroauricular Kimura Disease in a Young European Female: A Rare Case and Review of the Literature

**DOI:** 10.3390/life16010090

**Published:** 2026-01-07

**Authors:** Mircea Sorin Ciolofan, Ionuț Tănase, Carmen Aurelia Mogoantă, Daniel Pirici, Ilona Mihaela Liliac, George G. Mitroi, Loredana Elena Stoica

**Affiliations:** 1 Department of Otorhinolaryngology, Faculty of Medicine, University of Medicine and Pharmacy of Craiova, 200349 Craiova, Romania; sorin.ciolofan@umfcv.ro (M.S.C.);; 2 Department of Otorhinolaryngology, “Carol Davila” University of Medicine and Pharmacy, 020021 Bucharest, Romania; 3 Department of Pathology, University of Medicine and Pharmacy of Craiova, 200349 Craiova, Romania; 4 Department of Dermatology, Faculty of Medicine, University of Medicine and Pharmacy of Craiova, 200349 Craiova, Romania

**Keywords:** Kimura disease, retroauricular mass

## Abstract

**Background:** Kimura disease (KD) is a rare benign disorder usually presenting in young Asian males as a subcutaneous mass in the head and neck. Common histological findings include lymphoid follicular hyperplasia, eosinophilic infiltrates, and vascular proliferation. Non-endemic presentations, particularly in women, are rare. **Methods:** We report a case of isolated retroauricular KD in a 28-year-old White woman with a 3-year history of an isolated, enlarging, mildly painful retroauricular mass, accompanied by peripheral eosinophilia and elevated serum immunoglobulin E (IgE) levels. The mass was resected, imaging showed no other sites of concern, and there was no recurrence. **Results:** Histopathologically, eosinophilic microabscesses, prominent vascular proliferation, and lymphoid follicular hyperplasia with CD20^+^ B cells, CD3^+^ T cells, and preserved CD23^+^ follicular dendritic networks were identified. **Conclusions:** A diagnosis of angiolymphoid hyperplasia with eosinophilia (ALHE) was excluded, and a final diagnosis of KD was established.

## 1. Introduction

Kimura’s disease (KD) was initially reported in 1937 as an eosinophilic hyperplastic lymphogranuloma [1], but was more comprehensively described clinically and pathologically in 1948 by Kimura and Ishikawa [2]. Over 75% of cases present as a painless, poorly demarcated subcutaneous swelling in the head and neck region. Enlarged lymph nodes are common [3]. Systemic symptoms are rare. Pruritus, eczematous skin lesions, or other dermatologic manifestations may be seen [4,5,6,7,8,9]. Fever, weight loss, or night sweats are infrequent [9,10,11]. Elevated serum IgE levels (>90%) and moderate to marked eosinophilia are among the most characteristic KD findings. Inflammatory markers such as C-reactive protein (CRP) or erythrocyte sedimentation rate (ESR) may be elevated [12,13]. Some patients with KD develop renal involvement, with proteinuria or nephrotic syndrome; biopsies have revealed a spectrum of histopathological changes, including minimal change disease, focal glomerulosclerosis (FSGS), membranous nephropathy, IgA nephropathy, and membranoproliferative glomerulonephritis (MPGN) [14,15].

Definitive diagnosis relies on histopathology to distinguish KD from diagnoses such as ALHE, lymphoma, and reactive lymphadenopathy.

## 2. Case Report

**Figure 1:**
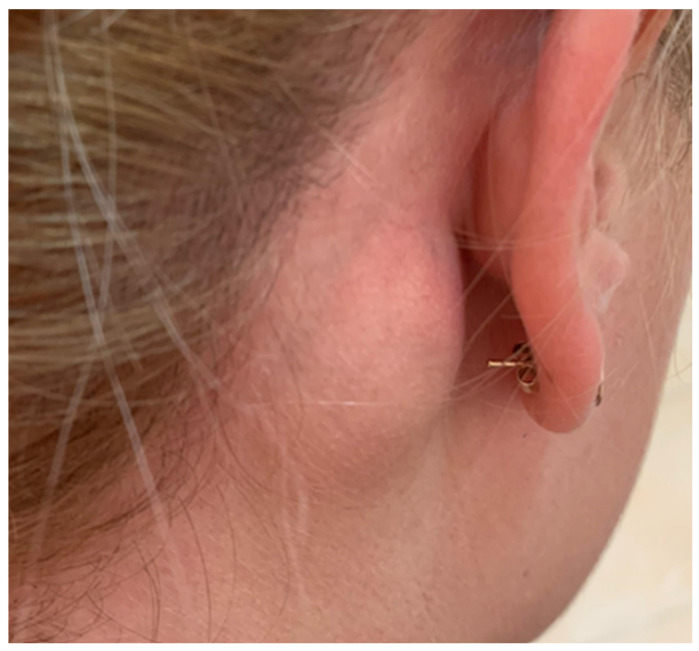
Subcutaneous mass located in the right retroauricular region.

A 28-year-old woman with no significant past medical or travel history presented with a three-year history of a mildly painful, mildly tender, enlarging ovoid 3 cm subcutaneous right retroauricular mass (Figure 1) with intact overlying skin, which had recently become tender and was the sole finding on complete physical examination. She reported mild night sweats and approximately 4 kg of weight loss, without other systemic symptoms.

Complete blood count showed eosinophilia, with an eosinophil count of 2.8 × 10^9^/L (normal <0.5 × 10^9^/L). Serum IgE level was 2500 IU/mL (normal <100 IU/mL). The C-reactive protein (CRP) level was 32 mg/L (normal <5 mg/L), and the erythrocyte sedimentation rate (ESR) was 52 mm/h (normal <20 mm/h). Liver and renal function tests were normal. Antinuclear antibodies (ANA), rheumatoid factor (RF), and anti-neutrophil cytoplasmic antibodies (ANCA) were negative, as were serologies for human immunodeficiency virus (HIV) and hepatitis B and C. Neck ultrasound and computed tomography (CT) showed no evidence of parotid gland involvement or additional sites of disease.

**Figure 2:**
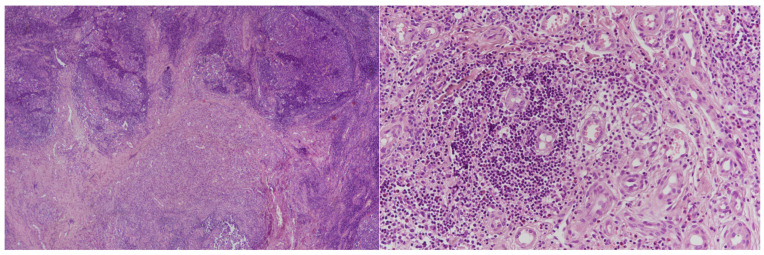
Dense lymphoid infiltrates with well-formed germinal centers, associated eosinophilic microabscesses, and a prominent capillary-rich vascular proliferation lined by plump endothelial cells. **Left**: H&E, ×10 magnification. **Right**: H&E, ×20 magnification.

The uncomfortable mass was uneventfully removed under local anesthesia. In the present case, excisional biopsy was favored over fine-needle biopsy because cytological evaluation is frequently non-diagnostic in KD, and complete excision allowed both definitive diagnosis and curative treatment of a localized, symptomatic lesion. In addition, the superficial location and cosmetic visibility of the mass supported a definitive surgical approach. Hematoxylin-and-eosin (H&E) staining showed dense lymphoid infiltrates with prominent germinal centers, eosinophilic microabscesses, and a rich vascular network of capillary-sized vessels lined by plump endothelial cells (Figure 2, Left and Right).

**Figure 3:**
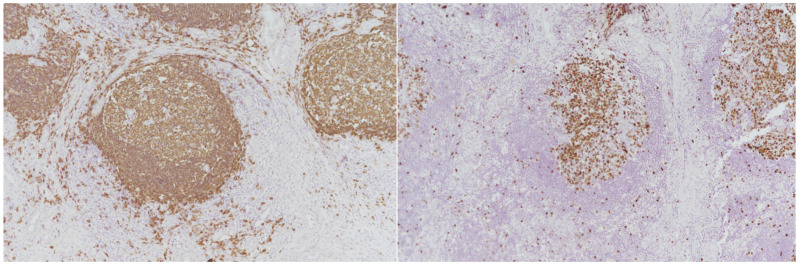
**Left**: lymphoid proliferation, with CD20^+^ B-cell follicles (×10 magnification); **Right**: Ki-67 staining (×10 magnification).

The inflammatory infiltrate extended into the surrounding soft tissue and was composed predominantly of small lymphocytes, eosinophils, and scattered plasma cells. IHC confirmed the polyclonal nature of the lymphoid proliferation, with CD20^+^ B-cell follicles, CD3^+^ T-cell zones, and preserved CD23^+^ follicular dendritic networks (Figure 3 Left).

Ki-67 staining showed a high proliferative index within the germinal centers, and no cytological atypia or malignancy was identified (Figure 3 Right).

The postoperative course was uneventful. The surgical site healed without complications, and the patient reported no pain, swelling, or recurrence of symptoms during the follow-up period. She was discharged with recommendations for outpatient surveillance.

To date, the patient remains in good clinical condition, with no evidence of local recurrence or new lesion development. The patient was followed clinically for approximately one year, during which no local recurrence or new lesions were identified.

## 3. Discussion

KD most frequently affects Asians aged 20 to 50 years, with a male-to-female ratio ranging from 3.5:1 to 9:1 [16]. Although the disease is usually seen in the head and neck region, particularly the parotid and submandibular areas, other locations (e.g., axilla, orbit, gingiva, and epiglottis) have been described. More widespread disease may include renal involvement, with proteinuria or nephrotic syndrome [17,18].

KD can be locally aggressive, and recurrences are common. Higher recurrence rates have been reported in association with larger lesion size, long-standing disease prior to diagnosis, marked peripheral eosinophilia, and elevated serum IgE levels [8]; however, most studies include few cases.

The histopathological hallmark of KD includes lymphoid follicular hyperplasia with enlarged germinal centers, a dense eosinophilic infiltrate, and the presence of eosinophilic microabscesses. These features are frequently accompanied by the proliferation of capillaries and small veins, often surrounded by fibrosis and collagen deposition [3]. Laboratory findings often, but not always, include serum eosinophilia and elevated serum IgE levels; however, these are not essential for diagnosis.

Although the etiology of KD is unknown, current evidence suggests that T helper (Th)2-mediated immune responses are involved in disease pathogenesis [7]. Elevated levels of Th2-associated cytokines, such as interleukin (IL)-4, IL-5, and IL-13, have been implicated in promoting IgE production, eosinophil proliferation, and tissue infiltration. These cytokines enhance chemokine signaling pathways that facilitate eosinophil migration into affected tissues. Studies have shown that individuals with KD exhibit a higher Th2/Th1 cell ratio and increased expression of Th2 cytokines in both blood and tissue, correlating with elevated serum IgE levels. Additionally, KD lesions demonstrate abundant Th2 cells, IgE, and IL-4-positive mast cells, supporting a dominant Th2-driven pathogenesis [19,20].

Excision is often the initial approach, both therapeutically and diagnostically. Other treatment options (e.g., irradiation, systemic steroids, and immunosuppressive or cytotoxic agents) have been employed alone or in combination, with varying degrees of success [21,22,23]. A 2022 meta-analysis of 31 papers including 44 cases (of which 70% underwent surgery alone) reported recurrence rates of 31% for surgical resection, 45% for immunosuppression, and 60% for irradiation; combined strategies were sometimes used. The overall recurrence rate was 24% [7].

We conducted a focused literature search in PubMed using the following search strategy: (“Kimura disease”) AND (“retroauricular” OR “postauricular” OR “auricular” OR “neck”). We limited the search to articles in English and screened abstracts to identify case reports and case series describing KD with retroauricular involvement. A full-text review was conducted to confirm the inclusion criteria. We included only reports that detailed clinical, histopathological, and therapeutic features of lesions anatomically centered in the retroauricular area. Articles were excluded if they lacked clinical detail, focused exclusively on renal or systemic manifestations without local lesion description, or involved the parotid gland with only secondary retroauricular extension. A formal PRISMA diagram was not included, given the small number of relevant case reports.

The 2025 comprehensive review by Lagerstrom [24] highlights that most KD cases in the head and neck involve the salivary glands or regional lymph nodes, with retroauricular involvement typically occurring as an extension from adjacent structures such as the parotid gland. The review also details key clinical and histopathological features of KD and its differential diagnoses, which may include several lymphomas, epithelioid hemangioma, Langerhans cell histiocytosis, reactive lymphadenopathy, and IgG4-related disease, in addition to ALHE.

In contrast to the case reported by Galib [25], which confirmed ALHE based on characteristic vascular endothelial proliferation, and the case by Itamura [26], which involved KD with extension toward the parotid gland, our patient presented with a strictly retroauricular lesion lacking significant endothelial atypia. Several previously reported cases of KD with postauricular involvement provide a useful framework for comparison. Gao [27] and Rathore [28] reported masses affecting both pre- and postauricular regions, often extending into the parotid or cervical nodes. Sherpa et al. [29] emphasized the diagnostic challenges posed by overlapping cytological features between KD and conditions such as tuberculosis and reactive lymphadenopathy, highlighting the essential role of histopathology.

Similarly, our case initially raised differential considerations, including ALHE; however, immunohistochemical findings (CD10^+^, Bcl-6^+^, Bcl-2^−^ in follicles, preserved CD23 meshwork, and high Ki-67 proliferation index) and the clinical context confirmed a reactive germinal center pattern consistent with KD and excluded ALHE. This distinction is particularly relevant given the case reported by Gupta [30], who described a similar postauricular presentation ultimately diagnosed as ALHE, reinforcing the need for thorough histological and immunophenotypic assessment. Nelson’s [31] early documentation of KD emphasized its histological hallmark of follicular hyperplasia with eosinophilic infiltration, a pattern echoed in our specimen. Thus, despite histological overlap with conditions such as ALHE or reactive lymphadenitis, immunohistochemistry and clinical correlation played a decisive role in achieving diagnostic clarity. A summary of these cases is presented in Table 1. Based on the available data, KD in Caucasian patients does not appear to differ significantly in clinical behavior or outcome compared with cases reported in Asian populations, although the number of reported non-Asian cases remains limited.

The differential diagnosis between KD and ALHE has long been debated; however, growing histopathological and clinical distinctions reinforce their classification as separate entities. In our patient, several key features strongly favored KD. Clinically, the lesion was a solitary, subcutaneous mass with normal overlying skin, typical of KD and in contrast to ALHE, which usually presents as multiple erythematous dermal nodules. Laboratory workup showed significant peripheral eosinophilia and markedly elevated serum IgE levels, both hallmarks of KD, while these findings are typically absent or mild in ALHE [32]. Histologically, our case revealed preserved lymph node architecture, florid lymphoid follicular hyperplasia with prominent germinal centers, eosinophilic microabscesses, and dense eosinophilic infiltrates—features characteristic of KD but not ALHE, which is instead marked by prominent vascular proliferation with hypertrophic, dome-shaped endothelial cells and sparse lymphoid follicle formation. These differences are well documented by Zou [32], who demonstrated that eosinophilic abscesses and germinal center hyperplasia distinguish KD from ALHE. Therefore, integration of clinical, laboratory, and histopathological data provides compelling evidence supporting the diagnosis of KD rather than ALHE.

## 4. Conclusions

KD presenting as a solitary retroauricular lesion is uncommon, and KD is rare in a young European woman. The histopathological and IHC findings, together with elevated serum IgE levels and eosinophilia (and mild systemic symptoms), established the diagnosis and excluded ALHE.

## Figures and Tables

**Table 1:** Clinical presentation, treatment, and outcomes of published cases of KD. NR, not reported.

Reference	Age/Sex/Ethnicity	Location	Laboratory Findings	Pathology/IHC	Treatment	Outcome
[25]	Adult/NR/NR	Postauricular	NR	Prominent vascular endothelial proliferation consistent with ALHE	Excised	Diagnosed as ALHE
[26]	Adult/NR/Asian	Postauricular with parotid extension	Eosinophilia, ↑ IgE	Typical KD features with salivary gland involvement	Excised ± steroids	No recurrence reported
[27]	Adult/NR/Asian	Pre- and postauricular, neck	Eosinophilia, ↑ IgE	KD with lymphoid follicular hyperplasia and eosinophilic infiltrates	Excised	Favorable
[28]	49/Male/Asian	Pre- and postauricular	Eosinophilia	Follicular hyperplasia with eosinophilic infiltrates	Steroids	Reduction in mass
[29]	Adult/NR/Asian	Postauricular	Eosinophilia	KD confirmed on histopathology; diagnostic difficulty noted	Excised	NR
[30]	Adult/NR/Asian	Postauricular	NR	Histology favored ALHE over KD	Excised	Diagnosed as ALHE
[31]	Adult/NR/NR	Postauricular	NR	Classic follicular hyperplasia with eosinophilic infiltration	NR	NR
Present case	28/Female/White	Strictly retroauricular	Eosinophilia, ↑ IgE	KD with eosinophilic microabscesses, preserved CD23 meshwork; ALHE excluded	Excised	No recurrence

↑ indicates increased/elevated levels compared with normal reference values.

## Data Availability

The original contributions presented in the study are included in the article, further inquiries can be directed to the corresponding author.

## Abbreviations

The following abbreviations are used in this manuscript:

KD	Kimura disease
IgE	immunoglobulin E
ALHE	angiolymphoid hyperplasia with eosinophilia
CRP	C-reactive protein
ESR	erythrocyte sedimentation rate
FSGS	focal segmental glomerulosclerosis
MPGN	membranoproliferative glomerulonephritis
ANA	antinuclear antibodies
RF	rheumatoid factor
ANCA	anti-neutrophil cytoplasmic antibodies
HIV	human immunodeficiency virus
HBV	hepatitis B virus
HCV	hepatitis C virus
CT	computed tomography
H&E	Hematoxylin and Eosin
IHC	immunohistochemical/immunohistochemistry
Th	T helper
IL	interleukin
CBC	complete blood count
WBC	white blood cell
ALT	alanine aminotransferase
AST	aspartate aminotransferase
eGFR	estimated glomerular filtration rate

## References

[1] Wang D., Mao J., Zhang Y., Gu W., Zhao S., Chen Y., Liu A. (2009). Kimura disease: A case report and review of the Chinese literature. Nephron Clin. Pract..

[2] Zhang G., Li X., Sun G., Cao Y., Gao N., Qi W. (2020). Clinical analysis of Kimura’s disease in 24 cases from China. BMC Surg..

[3] Meningaud J.-P., Pitak-Arnnop P., Fouret P., Bertrand J.-C. (2007). Kimura’s disease of the parotid region: Report of 2 cases and review of the literature. J. Oral Maxillofac. Surg..

[4] Chen H., Thompson L.D.R., Aguilera N.S.I., Abbondanzo S.L. (2004). Kimura disease: A clinicopathologic study of 21 cases. Am. J. Surg. Pathol..

[5] Zhang Y., Bao H., Zhang X., Yang F., Liu Y., Li H., Lu J., Liu Y. (2022). Kimura’s disease: Clinical characteristics, management and outcome of 20 cases from China. Clin. Exp. Rheumatol..

[6] Sangwan A., Goyal A., Bhalla A.S., Kumar A., Sharma R., Arava S., Dawar R. (2022). Kimura disease: A case series and systematic review of clinico-radiological features. Curr. Probl. Diagn. Radiol..

[7] Lee C.-C., Feng I.-J., Chen Y.-T., Weng S.-F., Chan L.-P., Lai C.-S., Lin S.-D., Kuo Y.-R. (2022). Treatment algorithm for Kimura’s disease: A systematic review and meta-analysis of treatment modalities and prognostic predictors. Int. J. Surg..

[8] Varshney M.K., Kumar A., Khan S.A., Yadav C.S. (2008). Kimura disease of extremity: Unusual manifestation in a long bone. Jt. Bone Spine.

[9] Zhang X., Jiao Y. (2019). The clinicopathological characteristics of Kimura disease in Chinese patients. Clin. Rheumatol..

[10] Molla Y.D., Alemu H.T., Zegeye K.B., Bekele T., Tadesse A.K., Answar I.O. (2024). Kimura disease, a rare Ethiopian case report. Heliyon.

[11] Alsmoudi H., Sleiay M., Almohamed A., Hamsho S., Alhadla A., Alqreea M., Alakhras A. (2024). A 23-year-old male patient with Kimura’s disease without renal transplantation: A rare case report from Syria. Ann. Med. Surg..

[12] Luo R., Yang G., Shi H., He Y., Han Y., Tian Z., Wu Y. (2025). A stepwise decision tree model for differential diagnosis of Kimura’s disease in the head and neck. BMC Med. Imaging.

[13] Park S.-W., Kim H.-J., Sung K.J., Lee J.H., Park I.S. (2012). Kimura disease: CT and MR imaging findings. AJNR Am. J. Neuroradiol..

[14] Liu Y., Liu S., Xu J., Xu X., Wang M. (2023). An unusual case of systemic lymphadenopathy—Kimura’s disease. J. Inflamm. Res..

[15] Gong Y., Gu J.-Y., Labh S., Shi Y.-L. (2015). Kimura disease accompanied with nephrotic syndrome in a 45-year-old male. Diagn. Pathol..

[16] Yang B., Liao H., Wang M., Long Q., Zhong H., Luo L., Liu Z., Cheng X. (2022). Kimura’s disease successively affecting multiple body parts: A case-based literature review. BMC Ophthalmol..

[17] Ray V., Boisseau-Garsaud A.M., Hillion G. (2003). Maladie de Kimura à localisation palatine chez un Antillais [Kimura’s disease on the hard palate in a patient from Martinique]. Rev. Med. Interne.

[18] Rajpoot D.K., Pahl M., Clark J. (2000). Nephrotic syndrome associated with Kimura disease. Pediatr. Nephrol..

[19] Ohta N., Fukase S., Suzuki Y., Ito T., Yoshitake H., Aoyagi M. (2011). Increase of Th2 and Tc1 cells in patients with Kimura’s disease. Auris Nasus Larynx.

[20] Yamazaki K., Kawashima H., Sato S., Tsunoda H., Yoshimura Y., Higuchi M., Hokibara S., Yamazaki T., Agematsu K. (2013). Increased CD45RO⁺ CD62L⁺ CD4⁺ T-cell subpopulation responsible for Th2 response in Kimura’s disease. Hum. Immunol..

[21] Abuel-Haija M., Hurford M.T. (2007). Kimura disease. Arch. Pathol. Lab. Med..

[22] Ye P., Ma D.Q., Yu G.Y., Gao Y., Peng X. (2017). Comparison of the efficacy of different treatment modalities for Kimura’s disease. Int. J. Oral Maxillofac. Surg..

[23] Lee D.N., Yeom S., Lee D.H., Lim S.C. (2024). Kimura Disease of the Head and Neck Region. Ear Nose Throat J..

[24] Lagerstrom I.T., Danielson D.T., Muir J.M., Foss R.D., Auerbach A., Aguilera N.S. (2025). A Comprehensive Review of Kimura Disease. Head Neck Pathol..

[25] Galib R., Gupta N., Rahman A., Aftab M., Qadri S., Alam K. (2024). An Unusual Presentation of Angiolymphoid Hyperplasia with Eosinophilia as Postauricular Mass: A case Report. Indian. J. Otolaryngol. Head Neck Surg..

[26] Itamura K., Swanson M. (2020). A Painless Retroauricular Mass. JAMA Otolaryngol. Head Neck Surg..

[27] Gao Y., Yan H., Zhong Y. (2025). A Case of Kimura Disease in the Left Postauricular and Neck Region. Ear Nose Throat J..

[28] Rathore A.V., Nagpure P. (2023). Common Postauricular Swelling Diagnosed as rare Kimura Disease. Indian. J. Otolaryngol. Head Neck Surg..

[29] Sherpa M., Lamichaney R., Roy A.D. (2016). Kimura’s disease: A diagnostic challenge experienced with cytology of postauricular swelling with histopathological relevance. J. Cytol..

[30] Gupta A., Shareef M., Lade H., Ponnusamy S.R., Mahajan A. (2019). Kimura’s Disease: A Diagnostic and Therapeutic Challenge. Indian. J. Otolaryngol. Head Neck Surg..

[31] Nelson S.M., Meyers A.D. (1978). Postauricular Kimura’s disease. Otolaryngology.

[32] Zou A., Hu M., Niu B. (2021). Comparison between Kimura’s disease and angiolymphoid hyperplasia with eosinophilia: Case reports and literature review. J. Int. Med. Res..

